# Management of microaspiration and gastrointestinal dysfunction after lung transplantation: A narrative review

**DOI:** 10.1016/j.jhlto.2025.100363

**Published:** 2025-08-08

**Authors:** René Hage, Carolin Steinack, Macé M. Schuurmans

**Affiliations:** a Division of Pulmonology, University Hospital Zurich, 8091 Zurich, Switzerland; b Faculty of Medicine, University of Zurich, 8032 Zurich, Switzerland

**Keywords:** Chronic lung allograft dysfunction, Bile acids, Pepsinogen A4, Gastroesophageal reflux, Anti-reflux surgery

## Abstract

**Background:**

Chronic Lung Allograft Dysfunction (CLAD) is the leading cause of late morbidity and mortality following lung transplantation. Increasing evidence implicates microaspiration, often secondary to gastroesophageal reflux disease (GERD) and gastrointestinal (GI) dysfunction, as a critical non-alloimmune driver of CLAD. However, its often silent presentation, diagnostic complexity, and heterogeneous management contribute to persistent knowledge and treatment gaps.

**Methods:**

This narrative review synthesizes recent literature on the pathophysiology, diagnosis, and clinical impact of microaspiration and GI dysfunction in lung transplant recipients. We focus on emerging biomarkers (e.g., conjugated bile acids and pepsinogen A4), diagnostic modalities, and both medical and surgical treatment strategies aimed at mitigating aspiration-induced graft injury.

**Key Content and Findings:**

Microaspiration leads to epithelial damage, surfactant disruption, immune activation, and microbial dysbiosis, collectively promoting allograft dysfunction. Conjugated bile acids in large airway bronchial wash fluid and pepsinogen A4 have shown superior specificity as aspiration biomarkers compared to pepsin alone. Gastrointestinal disorders, such as GERD, gastroparesis, and esophageal dysmotility, frequently co-exist post-transplant and contribute to aspiration risk. Pharmacologic interventions provide limited benefit, while anti-reflux surgery significantly improves graft outcomes, particularly when performed early. Conservative measures such as head-of-bed elevation also reduce reflux burden and may complement therapeutic strategies.

**Conclusions:**

Microaspiration is a modifiable and underrecognized contributor to allograft injury. Integration of aspiration biomarkers, early reflux evaluation, and personalized stepwise management, including surgical intervention when indicated, may improve long-term transplant outcomes. This review provides clinicians with a structured framework for diagnosis and management of microaspiration-related injury in lung transplantation.

## Introduction

### Background

Long-term survival in lung transplantation remains limited by the development of chronic lung allograft dysfunction (CLAD).^1^ While alloimmune injury has been the traditional focus of prevention, non-alloimmune factors such as microaspiration, are increasingly recognized contributors.^2^ International guidelines, including those from ISHLT/ATS/ERS, recommend objective reflux testing and early surgical referral in patients with suspected aspiration-related CLAD. However, clinical implementation remains inconsistent across centers.^3^ Microaspiration, defined as silent entry of gastric or duodenal contents into the lower airways, is closely linked to gastrointestinal (GI) dysfunction, particularly gastroesophageal reflux disease (GERD), which is often asymptomatic and can worsen post-transplant, with reported prevalence up to 65%.^4^, ^5^, ^6^, ^7^, ^8^, ^9^ While GERD describes retrograde flow into the esophagus, microaspiration refers to the entry of gastric contents into the lower airways. This distinction is important: not all GERD leads to microaspiration, but when it occurs, it may cause epithelial injury, local inflammation, and immune activation, that accelerates CLAD development.^10^ Despite its clinical importance, microaspiration remains difficult to diagnose and inconsistently managed, due to limited test accuracy and variability in clinical practice. This review outlines the pathophysiology, diagnostic challenges, and treatment strategies related to microaspiration and GI dysfunction in lung transplant recipients, with a focus on mitigating graft injury and improving outcomes.

### Methods

We performed a literature search in PubMed and Embase from 1 January 1990 to 15 May 2025 using the following keywords: “microaspiration” AND “lung transplantation” OR (“chronic lung allograft dysfunction” OR “CLAD” OR “bronchiolitis obliterans” OR “GERD” OR “pepsin” OR “pepsinogen A4” OR “gastroparesis” OR “esophageal dysmotility” OR “anti-reflux surgery” OR “pH impedance monitoring”). Additional references were identified through manual screening of reference lists in relevant studies and reviews. We included English-language original articles, reviews, and guideline papers focusing on the mechanisms, diagnosis, biomarkers, and treatment of aspiration-related lung injury post-lung transplantation. We did not perform a formal systematic review or meta-analysis, in line with the narrative review methodology.

### The pathophysiology of microaspiration in lung transplant recipients

**Figure 1:**
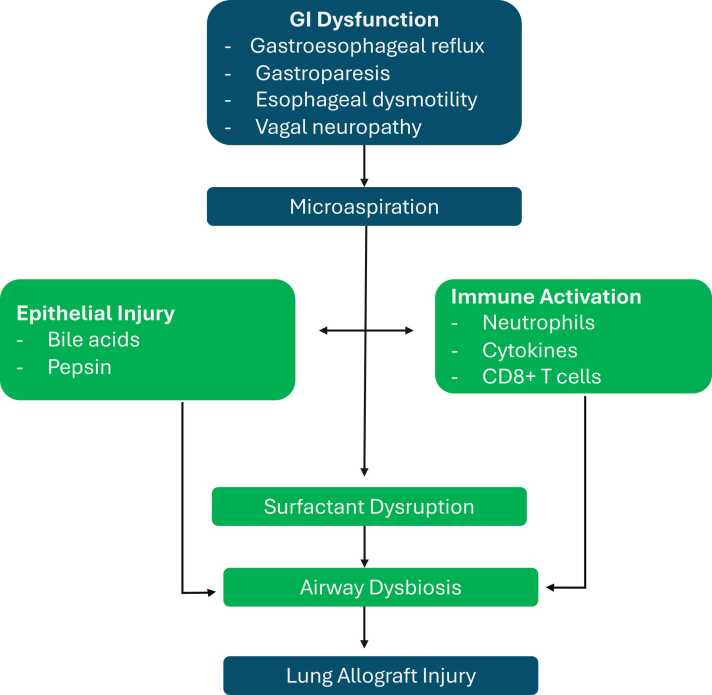
Proposed mechanisms linking gastrointestinal dysfunction to lung allograft injury, including epithelial damage, immune activation, and surfactant disruption induced by microaspiration.

Microaspiration is defined as the silent entry of gastroduodenal contents into the airways and has been linked to respiratory infections,^11^ acute cellular rejection,^12^ the formation of de novo donor-specific antibodies,^13^ CLAD,^14^ and reduced survival.^10^ Figure 1 summarizes the proposed pathophysiological mechanisms linking gastrointestinal dysfunction to microaspiration and subsequent lung allograft injury.

Its pathogenicity stems from components like bile acids, which are cytotoxic to lung tissue. Upon aspiration, they disrupt alveolar epithelium, destabilize surfactant, activate immune pathways, and promote fibrotic remodeling of the allograft parenchyma.^15^

Pulmonary surfactant, composed of phospholipids and proteins SP-A and SP-D, supports alveolar stability and innate defense. Bile acid aspiration disrupts this system by degrading surfactant lipids and inactivating SP-A/D, leading to impaired immunity, alveolar instability, and increased infection risk.^16^ Concurrently, bile acids induce neutrophilic alveolitis and upregulate cytokines such as interleukin (IL)−6, IL-8, IL-1β, and IL-10, which have pleiotropic effects on the immune response and may contribute to the recruitment of CD8+ T-cells and alloimmune activation.^15^ While CD8+ T cells may participate in graft injury via MHC class I-mediated mechanisms, cytotoxic activity typically requires activation through cytokine signaling and accessory cell interactions. Elevated bile acids in bronchoalveolar lavage (BAL) fluid correlate with CD8+ T cell infiltration and alloimmune activation(15).

Microaspiration may alter airway microbiota, facilitating colonization by pathogens such as *Pseudomonas aeruginosa* (PsA) which is associated with GERD and duodenogastric reflux.^17^ PsA can upregulate molecule B7 on neutrophils, activating CD4+ T cells, undermining allograft tolerance.^18^ In parallel, infection-driven expression of endothelin-1 (EC-1), promotes airway remodeling and fibrosis, accelerating CLAD.^19^

Microaspiration drives a multifactorial injury cascade involving epithelial damage, surfactant loss, immune activation, and microbiota-induced fibrosis. These mechanisms highlight that CLAD results from both alloimmune and aspiration-related injury.

### Diagnostic challenges and emerging biomarkers

Diagnosing microaspiration in lung transplant recipients is difficult due to its often silent and intermittent nature, often delaying detection until irreversible graft injury has occurred. Current diagnostics rely on BAL or large airway bronchial wash (LABW) to detect surrogate markers of gastric contents such as bile acids and pepsin.^10^ LABW refers to the instillation and aspiration of a small volume of sterile saline (typically 10–20 mL) into the trachea or mainstem bronchi during bronchoscopy, targeting secretions in the proximal airways where aspirated gastric contents initially deposit. In contrast, bronchoalveolar lavage (BAL) involves the instillation of larger volumes (usually 100–200 mL) into a wedged distal airway segment, resulting in dilution of airway lining fluid and potentially lower concentrations of aspiration biomarkers. As such, LABW may provide increased sensitivity for detecting conjugated bile acids and other surrogate markers of microaspiration.

Among these, elevated total bile acids, particularly the conjugated subtypes taurocholic acid (TCA) and glycocholic acid (GCA), have been associated with increased risk of CLAD.^15^, ^20^, ^21^, ^22^, ^23^, ^24^, ^25^ Recent evidence suggests that LABW is superior to BAL for detecting conjugated bile acids, as aspirated gastric contents initially deposit in the proximal airways.^25^ Accordingly, LABW TCA and GCA levels were found to be independently associated with acute lung allograft dysfunction and mortality, while bile acid levels in BAL were not predictive.^25^ These findings highlight the diagnostic and prognostic utility of conjugated bile acids measured in LABW samples. Moreover, given that bile acids in the lungs do not correlate with reflux burden measured by pH-impedance, direct sampling of the airways may provide more clinically relevant insights than traditional microaspiration testing.^25^

Bile acids are synthesized in the liver and typically conjugated with glycine or taurine before secretion into the duodenum. This conjugation enhances their solubility and limits passive reabsorption in the upper gastrointestinal tract. In the setting of small intestinal bacterial overgrowth, promoted by acid suppression therapies such as proton pump inhibitors (PPIs) or long-term macrolide use, bacterial enzymes can deconjugate bile acids. Consequently, the presence of unconjugated bile acids in the lungs may reflect altered gut microbiota rather than true aspiration. In contrast, conjugated bile acids, which originate directly from hepatic secretion, are more stable and specific markers of gastric content aspiration. Their detection in the lungs has been associated with increased airway inflammation, microbial dysbiosis, CLAD, and higher rates of hospitalization. Importantly, standard reflux therapies like PPIs and macrolides do not reduce total lung bile acid concentrations and may even shift the bile acid pool toward unconjugated forms.^24^ Thus, conjugated bile acids remain the most reliable biomarkers of enteric aspiration, while unconjugated forms may indicate microbiome-related changes under pharmacologic influence.^10^, ^24^, ^26^ Importantly, elevated lung bile acid levels, especially conjugated species, have also been linked to inflammatory cytokine profiles and adverse outcomes in non-transplant populations.^24^

Pepsin, though gastric-specific, is pH-sensitive and degrades in the alkaline lung environment, limiting its reliability.^27^, ^28^, ^29^, ^30^, ^31^ Moreover, pepsinogen C, also produced in the lung, may confound detection. In contrast, pepsinogen A4 (PGA4), a stomach-specific isoform, has emerged as a more accurate biomarker, and has shown promising results in distinguishing pulmonary versus gastric sources of pepsin-like activity.^14^ PGA4 can be detected with high specificity and has been shown to independently predict CLAD, supporting its use in surveillance bronchoscopies to identify high-risk patients.^14^

### Gastrointestinal dysfunction after lung transplantation

**Figure 2:**
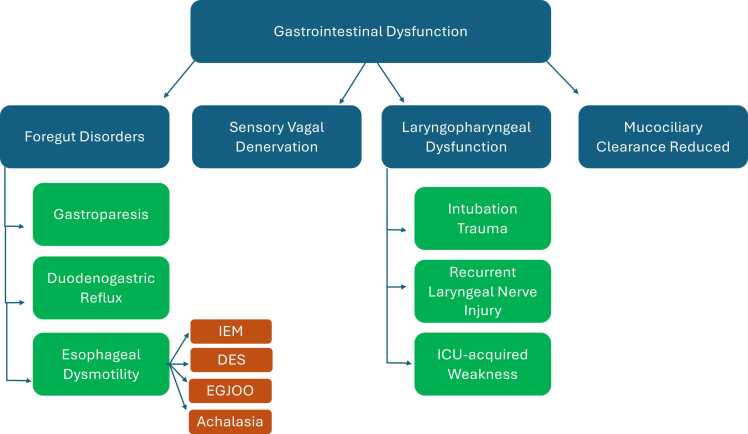
Spectrum of gastrointestinal dysfunction in lung transplant recipients contributing to microaspiration and CLAD.

Gastrointestinal (GI) dysfunction is common after lung transplantation and increasingly recognized as a driver of graft injury. A structured overview of common post-transplant GI disorders, their mechanisms, diagnostics, and therapeutic strategies is presented in Table 1. A graphical overview of the spectrum of gastrointestinal dysfunction is presented in Figure 2. Foregut disorders, including esophageal dysmotility, gastroparesis, and duodenogastric reflux, promote microaspiration and contribute to both acute and chronic allograft dysfunction. Airway protection is often compromised post-transplant due to altered airway and GI anatomy. Sensory vagal denervation impairs the cough reflex, increasing infection risk, while laryngopharyngeal dysfunction, due to intubation trauma, recurrent laryngeal nerve injury, or ICU-acquired weakness, leads to dysphonia, secretion retention, and oropharyngeal dysphagia.^32^ Additionally, mucociliary clearance is reduced to <15% of normal in transplanted lungs, exacerbating injury from persistent aspiration.^21^ Polymedication likely affects some of the above mechanisms.

**Table 1:** Overview of Gastrointestinal Dysfunction Subtypes After Lung Transplantation, Including Underlying Mechanisms, Diagnostic Tools, and Therapeutic Strategies

Dysfunction	Subtypes / Mechanisms	Diagnostic Tools	Therapeutic Options
GERD	Acid and non-acid reflux; transient LES relaxation; hiatal hernia	24-hour pH-impedance; endoscopy; airway biomarkers (pepsin, PGA4, bile acids)	PPIs (acid only), alginates, H2 receptor antagonists, lifestyle changes (e.g. head-of-bed elevation, weight loss), antireflux surgery (e.g. fundoplication)
Gastroparesis	Delayed gastric emptying	Gastric emptying scintigraphy; wireless motility capsule	Dietary modifications, prokinetics (e.g. metoclopramide, domperidone), gastric electrical stimulation, botulinum toxin injection, jejunal feeding, surgical options
Esophageal Dysmotility	IEM (ineffective esophageal motility), DES (diffuse esophageal spasm), EGJOO	High-resolution manometry	Prokinetics, calcium channel blockers (for DES), botulinum toxin, POEM, esophageal dilation, surgery in selected cases
Duodenogastric reflux	Bile acid reflux from duodenum into stomach and esophagus	24-hour pH-impedance; airway biomarkers (bile acids), gastroscopy, aspiration cytology	Prokinetics, bile acid sequestrants (e.g. cholestyramine), antireflux surgery
Vagal neuropathy	Impaired esophageal and gastric motility due to vagus nerve damage (post-transplant)	Gastric emptying tests, manometry, clinical suspicion	Supportive management, prokinetics, dietary adjustments, jejunal feeding

Abbreviations: DES = Distal esophageal spasm; EGJOO = Esophagogastric junction outflow obstruction; GERD = Gastroesophageal reflux disease; H2RA = Histamine-2 receptor antagonist; IEM = Ineffective esophageal motility; LARS = Laparoscopic antireflux surgery; LC-MS/MS = Liquid chromatography–mass spectrometry; LES = Lower esophageal sphincter; PGA4 = Pepsinogen A4; POEM = Peroral endoscopic myotomy; PPI = Proton pump inhibitor; TLESR = Transient lower esophageal sphincter relaxations

### Foregut motility disorders

#### Gastroesophageal reflux disease (GERD)

GERD is the most common foregut complication post-transplant, affecting up to 75% of recipients.^7^ It results from impaired lower esophageal sphincter (LES) tone, increased transient LES relaxations, delayed gastric emptying, and surgical factors such as esophagogastric junction (EGJ) disruption, vagal nerve injury or diaphragmatic dysfunction.^33^ Silent reflux is present in up to 50% and require objective testing, such as 24-hour pH-impedance monitoring.^34^ Non-acid reflux, particularly in proton pump inhibitor (PPI)-treated patients, may also drive aspiration-related injury.^29^ Persistent GERD is strongly associated with microaspiration and CLAD.^4^, ^10^, ^13^, ^29^, ^35^

#### Gastroparesis

Gastroparesis, defined as delayed gastric emptying without mechanical obstruction, affects 50–74% of lung transplant recipients.^36^, ^37^, ^38^, ^39^ It may be asymptomatic or present with nonspecific GI symptoms, such as nausea, bloating, early satiety, and vomiting.^37^ Causes include vagal nerve injury, opioid use, and immunosuppressants like cyclosporine (delaying motility) or mycophenolate mofetil (MMF), a prodrug of mycophenolic acid (MPA) causing GI side effects,^38^ while tacrolimus, a macrolide compound, may have mild prokinetic effects.^38^ In selected patients, switching to enteric-coated formulations of mycophenolate sodium (EC-MPS) improves tolerability, though the impact on true gastric motility remains uncertain.^40^ Autonomic dysfunction (AD) post- transplant may also contribute. AD can manifest with a wide range of symptoms due to impaired regulation of the autonomic nervous system, affecting the cardiovascular, gastrointestinal, and genitourinary systems. GI manifestations are particularly diverse and may include postoperative ileus, Ogilvie syndrome, gastroparesis^41^ or bowl perforation. Diagnosis is limited by underuse of gastric emptying scintigraphy (GES) and poor correlation with symptoms.^39^

#### Esophageal dysmotility

Post-transplant esophageal motility disorders, such as ineffective esophageal motility (IEM), distal esophageal spasm (DES), esophagogastric junction outflow obstruction (EGJOO), and achalasia, are increasingly recognized.^32^, ^42^ These impair esophageal clearance and increase aspiration risk, though their direct impact on graft survival remains unclear due to limited data.^32^ IEM, common in interstitial lung disease, involves weak peristalsis; DES causes premature, high-amplitude contractions with dysphagia or chest pain.^32^ EGJOO, associated with impaired LES relaxation, has been linked to CLAD in some studies.^32^ Rarely, achalasia may evolve from untreated EGJOO.^32^ Opioid-induced esophageal dysfunction (OIED) mimics these patterns but is reversible with opioid reduction or botulinum toxin.^32^

### Mechanisms linking foregut dysfunction to microaspiration

Foregut dysfunction contributes to microaspiration through impaired LES tone, delayed esophageal clearance and gastric stasis.^43^, ^44^, ^45^ Ineffective peristalsis and gastroparesis increase dwell time and proximal reflux, while EGJ abnormalities, particularly post-bilateral lung transplantation, further raise aspiration risk.^32^ These mechanisms act synergistically, promoting silent aspiration, epithelial injury, and alloimmune activation central to CLAD. Routine foregut function assessment is therefore essential, especially in patients with unexplained graft decline.

### Clinical evidence linking microaspiration to allograft outcomes

Microaspiration is a key contributor to allograft injury in lung transplantation, initiating epithelial cytotoxicity, immune activation, and surfactant disruption that drive both acute and chronic rejection. Gastrointestinal dysfunction has been strongly linked to the development of CLAD.^32^

### Biochemical and functional evidence

The presence of bile acids and pepsin in BAL fluid serves as a biochemical hallmark of aspiration. Elevated total bile acids are consistently associated with neutrophilic inflammation, early CLAD onset, reduced FEV1, and decreased overall survival.^21^, ^30^, ^31^, ^46^ Subtypes such as taurocholic and glycocholic acid have shown particularly strong predictive value.^30^, ^46^ Additional evidence from a prospective cohort study further strengthens the prognostic significance of bile acids in BAL.^22^ In this study, lung transplant recipients treated with azithromycin (AZM) for CLAD with bronchiolitis obliterans phenotype (BOS), who had detectable bile acids in BAL experienced a significantly more rapid FEV1 decline, faster progression to CLAD ≥ stage 1, and reduced 3-year survival compared to those without detectable bile acids.^22^

Similarly, high BAL pepsin concentrations are correlated with acute rejection and rapid pulmonary function decline.^30^, ^31^ Recent evidence further supports an association with primary graft dysfunction (PGD).^47^ In this prospective study of lung transplant recipients, pepsin concentrations in BAL were significantly elevated in patients with persistent grade 3 PGD (PGD-3) compared to those without PGD (PGD-0) and was detectable in nearly all PGD-3 samples but absent in healthy controls. Pepsin levels also correlated with altered microbiome composition, particularly enrichment in anaerobic taxa such as *Prevotella*, and with heightened inflammatory cytokine responses within the allograft.^47^ Notably the *Prevotella*/*Streptococcus* ratio emerged as a strong predictor of both pepsin concentration and PGD risk, suggesting a distinct microbiome signature associated with aspiration-related injury. These findings highlight the potential of pepsin, in combination with microbial and immune markers, as a biomarker for early allograft injury due to microaspiration.

In addition to being a useful biomarker of gastric aspiration, pepsin may also guide therapeutic strategies. Fisichella et al. investigated whether laparoscopic antireflux surgery could reduce pepsin levels in BAL fluid of lung transplant recipients.^28^ They found that patients with untreated GERD had significantly higher BAL pepsin concentrations compared to those who had undergone laparoscopic antireflux surgery, while healthy controls showed no detectable pepsin.^28^ Moreover, detectable pepsin levels were associated with faster progression to CLAD-BOS and more frequent episodes of acute rejection.^28^ These findings support the hypothesis that pepsin not only indicates aspiration but may also play a direct or indirect pathogenic role in allograft injury. Importantly, laparoscopic antireflux surgery appeared effective in mitigating this risk, suggesting that surgical reflux control might reduce microaspiration-related injury and improve long-term graft outcomes.^28^

Although pepsin is commonly measured in BAL to detect microaspiration, its specificity is limited by lung-derived pepsinogens. PGA4, a stomach-specific isoform, offers improved accuracy in LABW.^14^ Detectable PGA4 levels correlate with conjugated bile acids, airway infections, and increased risk of CLAD.^14^ Furthermore, PGA4 levels decreased after anti-reflux surgery, suggesting its potential as a dynamic biomarker for both diagnosis and monitoring of aspiration. In contrast, pepsin levels alone were not predictive of CLAD, potentially due to antibody cross-reactivity with lung-derived pepsinogens.^14^ These findings suggest that LABW PGA4 may outperform traditional BAL pepsin in identifying clinically relevant microaspiration and guiding post-transplant reflux management strategies.^14^

### GERD and CLAD: clinical cohort data

Multiple large cohort studies have underscored the impact of GERD, which is frequently asymptomatic in lung transplant recipients. Patients with objectively confirmed GERD (via pH-impedance monitoring) showed increased CLAD incidence and higher rates of donor-specific antibody (DSA) formation at 6–12 months post-transplant.^12^ Another cohort confirmed that untreated GERD was independently associated with CLAD, whereas early antireflux surgery significantly improved CLAD-free survival.^35^ Laparoscopic fundoplication, whether performed pre- or post-CLAD diagnosis, has been identified as an independent predictor of survival benefit, particularly in younger patients and those with restrictive lung disease.^46^ Notably, patients with silent GER also benefited, indicating that clinical symptoms are poor predictors of aspiration risk.^46^, ^48^ Even modest elevations in esophageal acid exposure were associated with decreased FEV1.^48^ Over 90% of patients undergoing antireflux surgery showed FEV1 stabilization or improvement,^49^ supporting a low threshold for reflux evaluation and surgical referral.

### Delayed gastric emptying

DGE is increasingly recognized as an independent risk factor for graft rejection. In one study, lung transplant candidates with pretransplant DGE showed a higher incidence of acute cellular rejection, even without GERD.^50^ By promoting gastric stasis and proximal content migration, especially in supine or postprandial states, DGE facilitates aspiration. Importantly, many affected patients are asymptomatic.^50^

### Immunological implications and DSAs

Aspiration-related epithelial injury may promote alloantigen exposure, facilitating both humoral and cellular alloimmune responses. This mechanistic link is supported by clinical data: patients with GERD and concurrent BAL bile acids exhibit higher DSA formation rates,^4^, ^35^, ^46^ highlighting a connection between microaspiration and loss of graft tolerance.

### Pharmacologic contributions

Post-transplant pharmacotherapy may further contribute to GI dysmotility. Cyclosporine is associated with gastroparesis, while tacrolimus has variable prokinetic effects. Mycophenolate mofetil (MMF), often implicated in upper GI symptoms, may worsen motility and increase aspiration risk.^4^ These effects must be considered when assessing graft outcomes in the context of GI dysfunction.

### Therapeutic strategies to prevent microaspiration-related injury

#### Medical therapy

First-line GERD treatment post-transplant includes proton pump inhibitors (PPIs), which reduce acid but not reflux frequency or volume. Thus, non-acid reflux, a key driver of aspiration injury, remains unaddressed. Bile acids and pepsin remain harmful even at higher pH levels. Prokinetic agents may help, especially in gastroparesis or hypomotility. Metoclopramide is a well-known drug in this setting, though long-term use is limited by extrapyramidal side effects. Domperidone, may be better tolerated due to its poor central nervous system penetration. Erythromycin, a macrolide antibiotic with motilin receptor agonist activity, has been used off-label to promote gastric emptying in gastroparesis, however, its long-term use is limited by the development of tachyphylaxis and the risk of QT interval prolongation.^51^ Tacrolimus may offer mild prokinetic benefit despite its primary immunosuppressive role.

#### Surgical antireflux interventions

Anti-reflux surgery (ARS), particularly laparoscopic Nissen fundoplication, is the most established intervention to reduce acid and non-acid reflux in lung transplant recipients.^52^ A 2025 meta-analysis of 1011 patients showed ARS improves FEV₁, slows its decline, and reduces mortality risk, especially when performed before CLAD onset.^52^ Benefits were observed even in asymptomatic patients. ARS also reduced inflammatory markers like IL-1β and pepsin, supporting its protective role. Alternative procedures such as transoral fundoplication or magnetic sphincter augmentation lack robust data. In gastroparesis refractory to medical therapy, pyloric interventions may help. Given these findings, early evaluation for ARS should be considered in lung transplant recipients with documented GERD or unexplained FEV₁ decline, in line with ISHLT/ATS/ERS guidelines, recommending “referral to an experienced surgeon to be evaluated for potential fundoplication” of the gastro-esophageal junction in case of a decline in FEV1 consistent with the onset of CLAD BOS and a confirmed GERD.^3^

#### Supportive and preventive measures

Conservative strategies to reduce gastroesophageal reflux and microaspiration are crucial in lung transplant recipients due to their association with chronic lung allograft dysfunction. Among these, head-of-bed elevation (HOBE) has demonstrated clinical efficacy in reducing reflux symptoms and esophageal acid exposure. A systematic review including five controlled trials (n=228), reported consistent improvements in patient-reported reflux symptoms and intra-esophageal pH metrics with HOBE using 15–20 cm bed blocks or wedge pillows.^53^ In a high-quality crossover trial (n=65), HOBE led to a clinically meaningful reduction in symptom scores (RR 2.1, 95% CI 1.2–3.6) and was preferred by 63% of participants over flat sleeping.^54^ Acid exposure and reflux episodes are reduced by behavioral measures. We recommend elevating the head of the bed (15–20 cm), avoiding meals before sleep, limiting evening caffeine, and reducing weight if overweight. These low-risk interventions should be standard in post-transplant reflux management to prevent delayed gastric emptying and aspiration.

### Integration into transplant pathways

A structured, algorithmic approach, summarized in Table 2, can guide stepwise escalation from medical to surgical therapy based on reflux, delayed gastric emptying, or aspiration biomarkers. Although consensus guidelines advocate reflux screening and antireflux surgery in selected lung transplant recipients,^3^ implementation varies widely due to resource availability, institutional preferences, and lack of standardized pathways. Routine objective testing and multidisciplinary care should be integrated into transplant protocols, especially during the first post-operative year, to identify silent GI dysfunction and optimize graft outcomes.

**Table 2:** Diagnostic and Therapeutic Strategies for Selected Clinical Scenarios Associated with Gastrointestinal Dysfunction and Aspiration After Lung Transplantation

Clinical Scenario	Recommended Diagnostics	Key Findings / Biomarkers	Suggested Interventions
Routine surveillance (asymptomatic patient)	- 24-hour pH-impedance monitoring - LABW during surveillance bronchoscopy - Gastric emptying scintigraphy (if feasible)	- Elevated conjugated bile acids (e.g., TCA, GCA) - Detectable PGA4	- Lifestyle modifications (e.g., head-of-bed elevation, dietary adjustments) - Consider early antireflux surgery in high-risk profiles
Unexplained FEV₁ decline / suspected CLAD	- BAL or LABW for pepsin, PGA4, bile acids - High-resolution manometry - DSA screening	- Bile acids >10 nmol/L - Positive PGA4 - Abnormal reflux profile on impedance-pH	- Escalate medical therapy (PPI, prokinetics) - Strong consideration for antireflux surgery
Symptomatic GERD or gastroparesis	- Symptom assessment (e.g., reflux scores) - pH-impedance monitoring - Gastric emptying scintigraphy	- Delayed gastric emptying >10% retention at 4 h - Positive acid/non-acid reflux profile	- Prokinetics (e.g., domperidone, erythromycin) - Pyloric botulinum toxin, dietary modification - Fundoplication or pyloroplasty if refractory
Refractory CLAD or rapid progression	- Repeat BAL/LABW for biomarker trend - Consider ET−1 and airway microbiome profiling	- Persistent biomarker elevation - Ongoing airway inflammation despite treatment	- Surgical escalation if not already performed - Consider clinical trial enrollment or immunosuppressive adjustment

Abbreviations: BAL: Bronchoalveolar lavage; CLAD: Chronic lung allograft dysfunction; DSA: Donor-specific antibodies; ET-1: Endothelin-1; FEV₁: Forced expiratory volume in one second; GCA: Glycocholic acid; GERD: Gastroesophageal reflux disease; LABW: Large airway bronchial wash; PGA4: Pepsinogen A4; PPI: Proton pump inhibitor; TCA: Taurocholic acid.

## Conclusion

Although microaspiration is a conceptually Ccompelling and potentially modifiable contributor to CLAD, direct evidence remains limited. Current diagnostic modalities often rely on surrogate biomarkers, and microaspiration is frequently inferred rather than observed directly. In some cases, microaspiration may serve as an explanatory framework adopted in the absence of definitive alternatives. This reflects broader gaps in our understanding of CLAD pathogenesis and the need for refined, mechanism-based diagnostics.

Microaspiration is a treatable and clinically significant contributor to CLAD, caused by gastrointestinal dysfunction such as GERD, gastroparesis, and esophageal dysmotility. These conditions promote epithelial injury and immune activation but often remain underrecognized. Objective diagnostics and stepwise management, ranging from acid suppression to antireflux surgery, may prevent further graft damage. Structured algorithms may help standardize care and guide individualized treatment. Microaspiration should be viewed not as incidental but as a key therapeutic target to improve long-term outcomes after lung transplantation.

## Funding

This research did not receive any specific grant from funding agencies in the public, commercial, or not-for-profit sectors.

## CRediT authorship contribution statement

René Hage: Conceptualization, Investigation, Methodology, Writing original draft.

Carolin Steinack: Writing: review and editing, Validation.

Macé M. Schuurmans: Writing: review and editing, Validation, Resources, Supervision.

## Declaration of Generative AI and AI-Assisted Technologies in the Writing Process

Statement: During the preparation of this work, the authors used ChatGPT (developed by OpenAI) to assist with language editing and improving clarity. Following the use of this tool, the author(s) thoroughly reviewed and edited the content as necessary and take full responsibility for the integrity and accuracy of the final manuscript.

## Declaration of Competing Interest

The authors declare that they have no known competing financial interests or personal relationships that could have appeared to influence the work reported in this paper.
